# A Hybrid Forecasting Approach to Air Quality Time Series Based on Endpoint Condition and Combined Forecasting Model

**DOI:** 10.3390/ijerph15091941

**Published:** 2018-09-06

**Authors:** Jiaming Zhu, Peng Wu, Huayou Chen, Ligang Zhou, Zhifu Tao

**Affiliations:** 1 School of Mathematical Sciences, Anhui University, Hefei 230601, Anhui, China; 18355150818@163.com (J.Z.); littlepengwu@126.com (P.W.); shuiqiaozlg@126.com (L.Z.); 2 School of Economics, Anhui University, Hefei 230601, Anhui, China; zhifut_0514@163.com

**Keywords:** air quality index, hybrid forecasting approach, ensemble empirical mode decomposition, end effect, combined forecasting model

## Abstract

Air pollution forecasting plays a vital role in environment pollution warning and control. Air pollution forecasting studies can also recommend pollutant emission control strategies to mitigate the number of poor air quality days. Although various literature works have focused on the decomposition-ensemble forecasting model, studies concerning the endpoint effect of ensemble empirical mode decomposition (EEMD) and the forecasting model of sub-series selection are still limited. In this study, a hybrid forecasting approach (EEMD-MM-CFM) is proposed based on integrated EEMD with the endpoint condition mirror method and combined forecasting model for sub-series. The main steps of the proposed model are as follows: Firstly, EEMD, which sifts the sub-series intrinsic mode functions (IMFs) and a residue, is proposed based on the endpoint condition method. Then, based on the different individual forecasting methods, an optimal combined forecasting model is developed to forecast the IMFs and residue. Finally, the outputs are obtained by summing the forecasts. For illustration and comparison, air quality index (AQI) data from Hefei in China are used as the sample, and the empirical results indicate that the proposed approach is superior to benchmark models in terms of some forecasting assessment measures. The proposed hybrid approach can be utilized for air quality index forecasting.

## 1. Introduction

Air pollution has become an increasingly important issue in environmental sciences. The general public has become increasingly attentive to poor air quality forecasts due to the serious impact that pollution has on human health and its limitation on outdoor activities, especially in China [1,2,3]. Therefore, the development of advanced air pollution forecasting systems is an emerging topic for research studies.

Air pollution levels are assessed through various indicators. The AQI is the one that reflects air quality status. According to calculation outcomes under the new ambient air quality standards of China (GB3095-2012), it integrates multiple pollutants into a single numerical form covering six pollutants (sulfur dioxide (SO${}_{2}$), nitrogen dioxide (NO${}_{2}$), PM${}_{2.5}$, PM${}_{10}$, ozone (O${}_{3}$) and carbon monoxide (CO) [4]). Currently, AQI is a vital reference for outdoor activity decisions. It has six classes and provides suggestions on outdoor activity correspondingly for different people in terms of different physical qualities (Table 1). Therefore, it is necessary to develop an effective AQI forecasting model.

According to existing literature, abundant forecasting models, including atmospheric chemical transport forecasting models and data-driven forecasting models, have been proposed for air quality and the indicator AQI. The atmospheric chemical transport forecasting model is a forecasting system that provides a large-scale, offline, time-space continuous model that estimates intercontinental transport of atmospheric pollutants. The intercontinental transport results in North America and Europe can be obtained according to the atmospheric transport continuity equation [5,6]. However, the advantage of the data-driven forecasting model is that we can choose some statistical models or artificial intelligence models according to the characteristics of the data, and it is easy for us to solve them with software, while the disadvantage is how to select the appropriate factors to describe air quality prediction systems. This paper focuses on the prediction of AQI; thus, we mainly consider the data-driven forecasting models. Related data-driven forecasting models are classified into three main types: models using traditional statistical methods, models applying artificial intelligence techniques and models utilizing combined and hybrid forecasting approaches.

As for traditional statistical models, the auto-regressive integrated moving average (ARIMA) model, the automated correction technique, multiple linear regression (MLR), the principal component regression (PCR) technique and the non-parametric regression (NR) method have been widely applied to AQI and air quality forecasting. For instance, Reikard and Slini et al. utilized the ARIMA model to predict the AQI and air pollution [7,8]. Neal et al. developed an automated bias correction scheme for air quality forecasting, and the proposed model has good precision in the five days forecasted ahead [9]. Camillo et al. proposed a bias adjustment technique to improve air quality forecasting [10]. Goyal et al. used the MLR and PCR methods for air quality forecasting in Delhi, respectively [11,12]. Aoife et al. developed an NR model for hourly NO${}_{2}$ forecasting [13].

As for AI techniques, artificial neural networks (ANN) and support vector regression (SVR) might be the famous models for air quality forecasting. Jiang et al. [14], Hooyberghs et al. [15], Ordieres et al. [16], Voukantsis et al. [17], Elangasinghe et al. [18] and Feng et al. utilized ANN techniques for AQI and air pollution forecasting [19], respectively. Ortiz-García et al. and Yeganeh et al. applied the SVR algorithms to forecasting O${}_{3}$ and CO [20,21]. Wang et al. proposed a comprehensive warning system based on a modified least squares support vector machine and a cloud model, and the empirical results showed that the warning system yielded remarkably high performance and has been widely used [22]. Generally, the ANN is different from traditional statistical models. It is capable of modeling non-linear relationships between input and output variables and is often used in forecasting variables in complex systems. However, non-linear relationships described by the ANN model are unsuitable to present an analytic expression for the forecasting model.

Another suitable model for unstable and nonlinear time series is the hybrid forecasting approach, which integrates the EEMD algorithms [23] and single forecasting model. The main steps of the hybrid forecasting approach are as follows: firstly, employing the EEMD algorithms to sift the original data to obtain one group of smoother IMFs and a residue; then, utilizing the forecasting model for IMFs and the residue; at last, summing forecasts and obtaining outputs. Hybrid AI models are popular in practical application in the fields of crude oil price forecasting [24,25,26], wind speed forecasting [27], electrical load forecasting [28,29] and air quality forecasting. Zhu et al. proposed two hybrid models for daily AQI forecasting in Xingtai [30]. Niu et al. proposed a novel hybrid decomposition-and-ensemble model for PM_2.5_ based on complementary EEMD, the grey wolf optimizer and SVR [31]. Zhou et al. presented a general regression neural network (GRNN) model combining EEMD. In this research, the EEMD technique was exploited to decompose raw PM_2.5_ data into some IMFs and residues, and GRNN was implemented to forecast each IMF and residue series [32]. The empirical tests showed that hybrid AI models are more effective and robust than any single model.

However, on the one hand, despite the effectiveness and robustness of hybrid forecasting models based on EEMD, these approaches always neglect the endpoint effect. As discussed in [33,34], however, the two ends of a time series will disperse, while the series is decomposed by EEMD, and this dispersion, termed the end effect, would “empoison” the whole time series, gradually making the results distorted. To be more specific, the end effect occurs during the sifting process, when the endpoints cannot be identified as the extrema in the procedure of decomposition. Wu et al. proposed an improved method for restraining the end effect in empirical mode decomposition by using known points to extend both the beginning and end of the series [34]. On the other hand, the selection of a suitable forecasting model for sub-series is a tough problem. Combination forecasting was initially introduced by Bates and Granger [35]. It improves forecasting accuracy and reduces risk effectively; thus, it leads to wide application in social-economics, the eco-environment and management, etc. [36,37]. Therefore, to address these issues, a hybrid forecasting approach has been proposed with integrated EEMD based on the endpoint condition method and the combined forecasting model for sub-series forecasting. The main steps of the proposed model are as follows successively: applying EEMD with the endpoint condition method to sift the original AQI time series; considering that forecasting accuracy varies with time points and methods, constructing the optimal combined forecasting model by applying the induced ordered weighted averaging (IOWA) operator, summing the forecast outputs and testing the forecasting accuracy.

The primary contributions of this paper are described as follows:

- Based on the decomposition and ensemble strategy, the endpoint condition method is utilized to sift IMFs and residues.
- A hybrid forecasting approach is proposed based on the varied weight combined forecasting model and EEMD.
- Some evaluation measures and model test are employed to estimate the forecasting performance of the developed hybrid approach.
- The developed hybrid approach significantly improves the forecasting accuracy of AQI.

The structure of this study is organized as follows: Section 2 introduces the study city and dataset. Section 3 proposes several individual forecasting models and hybrid forecasting approaches. The forecasting results and performance are discussed in Section 4. Finally, conclusions and further research are discussed in Section 5.

## 2. Study Area and Dataset

**Figure 1:**
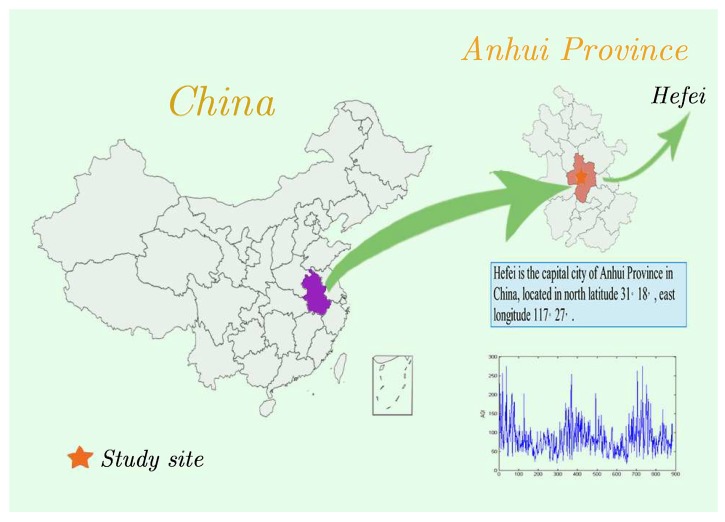
Geographic location of Hefei in China.

Hefei is the capital city of Anhui Province in China, located at north latitude $31^{\circ }$18${}^{\prime }$, east longitude $117^{\circ }$27${}^{\prime }$ (Figure 1). Hefei is an important national science and education center and the first national science and technology innovation city. Hefei is also one of the famous tourist cities. With the development of the economy, the air quality of Hefei is also getting worse, which poses a threat to people’s health and travel. Therefore, accurately forecasting the AQI index can provide good advice for people’s outdoor activities. In this study, the data of AQI were collected from the web sites http://www.zhb.gov.cn/ and http://www.tianqihoubao.com/aqi/hefei.html. We selected the dataset of daily AQI from 1 January 2016–31 May 2018 with a total of 884 observations; the mean was 85.00; the maximum was 275; the minimum was 17. The class of AQI in Hefei ranged from Class I–Class V (Figure 1). The sample data were partitioned into subsets, i.e., training set (1 January 2016–30 April 2018) and testing set (1 May 2018–30 May 2018).

## 3. Methodology

This section presents a hybrid forecasting approach with EEMD and an optimal combined forecasting model. In particular, Section 3.1 provides an overview of the proposed model, and Section 3.2, Section 3.3 and Section 3.4 introduce EEMD, the individual forecasting technique and the optimal combined forecasting model, respectively.

### 3.1. Overview of the Proposed Hybrid Methodology

**Figure 2:**
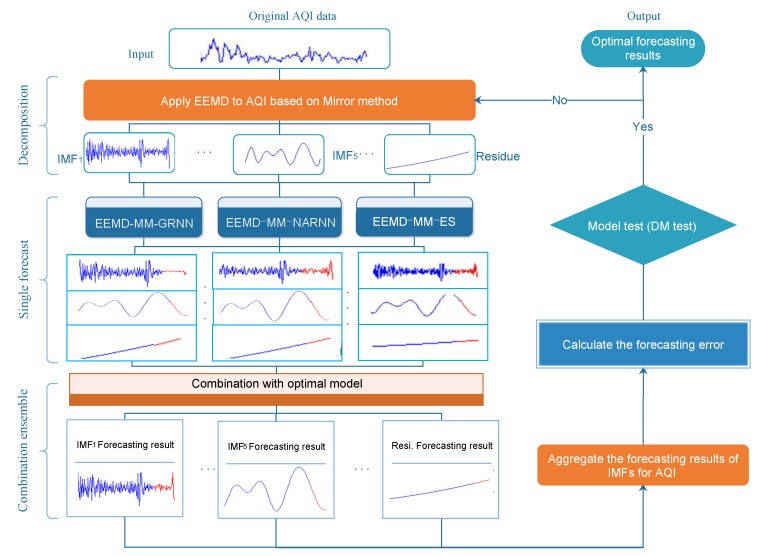
Framework of the proposed hybrid forecasting approach.

To enhance forecasting accuracy, a hybrid forecasting approach with EEMD based on the mirror method and the optimal combined forecasting model is proposed for AQI forecasting. We introduce the mirror method for eliminating the impact of the endpoints because the endpoints adversely affect the results of the decomposition by EEMD. Meanwhile, in order to address the difficult problem of how to choose the model for forecasting the IMFs and residue, we propose a varied weight combined forecasting model. The framework of the proposed hybrid forecasting approach is as illustrated in Figure 2.

The main steps of the proposed hybrid forecasting approach are as follows:

Step 1: Data decomposition.

The EEMD with mirror method is applied to decompose the original AQI data $x_{t}\left(t=1,2,\ldots ,N\right)$ into *N* IMFs, $c_{t}\left(k\right),\;\;\;k=1,2,\cdots K$, and one residue $r_{t}$.

Step 2: Individual forecast.

In this step, individual forecast techniques, such as the general regression neural network (GRNN) model, the nonlinear autoregressive neural network (NARNN) model and exponential smoothing (ES) method, are employed to forecast the IMFs and residue series. Accordingly, the forecasting results are denoted by $c_{it}\left(k\right)$ and $r_{it}$, respectively.

Step 3: Combined forecasting model for IMFs and residue

To improve accuracy and diversify the risk of forecasting effectively, the combined forecasting model is used to forecast IMFs and residue series by integrating different individual forecasting models mentioned above. Considering that forecasting accuracy varies with time points and methods, the IOWA ensemble operator is applied to construct the optimal combined forecasting model. The combined forecasting result can be written as ${\hat{c}}_{t}\left(k\right)=\sum_{i=1}^{m}w_{i}c_{a-index(it)}$ and ${\hat{r}}_{t}=\sum_{i=1}^{m}w_{i}r_{a-index(it)}$, respectively, where *m* is the total number of individual models, and the weight $w_{i}$ meets the conditions that $w_{i}\in \left[0,1\right],\sum_{i=1}^{m}w_{i}=1$.

Step 4: Ensemble forecast.

In this step, the forecast results of AQI can be obtained by summing ${\hat{c}}_{t}\left(k\right)$ and ${\hat{r}}_{t}$ with a simple addition approach. It can be described as:

(1) $${\hat{x}}_{t}=\sum_{k=1}^{K}{\hat{c}}_{t}\left(k\right)+{\hat{r}}_{t}.$$

Step 5: Model test and comparison.

The final outputs are obtained through the model test and comparison.

### 3.2. EEMD with the Endpoint Condition Method

#### 3.2.1. EEMD

The EEMD technique, first proposed by Huang et al., is a kind of adaptive signal decomposition technique using the Hilbert–Huang transform (HHT). The EEMD technique is an improvement of the EMD technique [38], and it can be employed to nonlinear and non-stationary time series. The EEMD technique decomposes the original series into IMFs and residue. The IMF is a function that satisfies two conditions:

1. In the whole dataset, the number of zeros and the number of extreme crossings must either be equal or differ at most by one;
2. At any point, the mean value of the envelope defined by the local maxima and local minima is zero.

The calculation steps of the EEMD technique for an original time series are listed in the following:

Step 1: In the original time series $x_{t}$, random white noise obeying a normal distribution is added to generate the new time series $y_{t}$.
Step 2: Let $i=0,\;y_{i,t}=y_{t}$, and calculate all of the local maxima and local minima.
Step 3: Interpolate the local maxima by a cubic spline to obtain upper envelop $h_{\max,i,t}$, and the lower envelop $h_{\min,i,t}$ can be obtained similarly.
Step 4: Compute the mean envelop: mi,t=(hmax,i,t+hmin,i,t)hmax,i,t+hmin,i,t)22.
Step 5: Let $r_{i,t}=y_{i,t}-m_{i,t}$, and judge whether $r_{i,t}$ meets the two conditions of IMFs. If it satisfies the two conditions, then $r_{i,t}$ is the *i*-th $IMF_{i,t}$; otherwise, let $y_{i,t}=y_{i-1,t}-r_{i-1,t}$, and repeat Step 2–Step 5.
Step 6: Repeat Step 2–Step 5 until the residue is a constant or trend time series.
Step 7: Based on different random white noise, repeat Step 1–Step 6 NE times; NE is the number of ensemble members.
Step 8: Find the ensemble and mean results from Step 7 to obtain the final result, i.e., the $IMF_{i,t}$ and the residue $r_{i,t}$.

#### 3.2.2. Endpoint Condition Method

In this study, we introduce a sample and effective method, i.e., the mirror method (MM). The MM uses the known points to extend both the beginning and end of the series. For the beginning of time series $x_{t}$, add local minimum Min(0) by mirror symmetry with respect to the local maximum Max(1); for the end of the time series, add local maximum Max(n+1) by mirror symmetry with respect to the local minimum Min(n). The newly obtained Min(0) and Max(n+1) are then taken for construction of the lower and upper envelopes along with initial extrema.

Just as mentioned above, the Algorithm 1, which incorporated EEMD technique and mirror method is depicted as follows.
**Algorithm 1** for data decomposition1: **procedure**
$x_{t}$.2:    **for**
1≤l≤NE
**do**3:       $y_{t}^{l}\leftarrow x_{t}+\varepsilon^{l}$, $y_{i=0,t}^{l}\leftarrow y_{t}^{l}$4:       $Max\_local\leftarrow \mathrm{local}\mathrm{maxima}\;y_{i,t}^{l}$, $Min\_local\leftarrow \mathrm{local}\mathrm{minima}\;y_{i,t}^{l}$5:       ${\bar{y}}_{i,t}^{l}\leftarrow$ apply endpoint condition method to $y_{i,t}^{l}$6:       ${\bar{h}}_{\max,i,t}^{l}\leftarrow$ upper envelop of ${\bar{y}}_{i,t}^{l}$; ${\bar{h}}_{\max,i,t}^{l}\leftarrow$ lower envelop of ${\bar{y}}_{i,t}^{l}$7:       ${\bar{m}}_{i,t}^{l}\leftarrow \frac{{\bar{h}}_{\max,i,t}^{l}+{\bar{h}}_{\min,i,t}^{l}}{2}$, ${\bar{r}}_{i,t}^{l}\leftarrow {\bar{y}}_{i,t}^{l}-{\bar{m}}_{i,t}^{l}$8:       **while**
${\bar{y}}_{i,t}^{l}$ is a constant or trend **do**9:       **if**
${\bar{r}}_{i,t}^{l}$ satisfies the two conditions of IMFs, **do**10:          ${\bar{r}}_{i,t}^{l}$ is the *i*-th $IMF_{i,t}^{*}$11:          i←i+112:          ${\bar{y}}_{i,t}^{l}={\bar{y}}_{i-1,t}^{l}-{\bar{r}}_{i-1,t}^{l}$13:       **else**14:          ${\bar{y}}_{i,t}^{l}={\bar{r}}_{i,t}^{l}$15:       **end if**16:       $Max\_local\leftarrow \mathrm{local}\mathrm{maxima}\;y_{i,t}^{l}$, $Min\_local\leftarrow \mathrm{local}\mathrm{minima}\;y_{i,t}^{l}$17:       ${\bar{h}}_{\max,i,t}^{l}\leftarrow$ upper envelop of ${\bar{y}}_{i,t}^{l}$; ${\bar{h}}_{\max,i,t}^{l}\leftarrow$ lower envelop of ${\bar{y}}_{i,t}^{l}$18:       ${\bar{m}}_{i,t}^{l}\leftarrow \frac{{\bar{h}}_{\max,i,t}^{l}+{\bar{h}}_{\min,i,t}^{l}}{2}$19:    **end while**20: **end for**21: **end procedure**
$IMF_{i,t}^{*}$ and ${\bar{r}}_{i,t}$

### 3.3. Individual Forecasting Model

#### 3.3.1. General Regression Neural Network Model

Specht [39] proposed a type of neural network model called a GRNN, which has strong nonlinearity mapping capacity and a flexible network structure.

A GRNN comprises four layers, i.e., an input layer, pattern layer, summation layer and output layer. The input and output vector can be described as: $X={\left(x_{1},x_{2}\cdots x_{n}\right)}^{T},Y={\left(y_{1},y_{2}\cdots y_{k}\right)}^{T}$ respectively.

(1) Input layer

The number of input layer neurons is equal to the dimension of the input vector. Then, the pattern layer is fed data from the input neurons of the input layer.

(2) Pattern layer

The number of neurons is equal to the number of training samples *n*. The pattern uses a nonlinear function, i.e., the Gaussian function of $p_{i}$ is described as:

(2) $$p_{i}=e^{-\frac{{\left(X-X_{i}\right)}^{T}\left(X-X_{i}\right)}{2\sigma^{2}}},\left(i=1,2,\cdots ,n\right).$$

(3) Summation layer

The summation layer utilizes two kinds of summations, the simple summation $S_{s}$ and the weighted summation $S_{wj}$. The transfer functions can be written as Equations (3) and (4):

(3) $$S_{s}=\sum_{t=1}^{n}p_{t},$$

(4) $$S_{wj}=\sum_{t=1}^{n}w_{t}p_{t},j=1,2,\cdots k,$$

where $w_{t}$ is the weight of pattern neuron *t* that is connected to the summation layer and $p_{t}$ is the outputs that belong to the pattern layer.

(4) Output layer

In the output layer, the number of neurons is equal to the dimension *k* of the output vector *Y*, and the forecasting results of neuron *j* can be computed as:

(5) $${\hat{Y}}_{j}=\frac{S_{s}}{S_{wj}},j=1,2,\cdots ,k.$$

#### 3.3.2. Nonlinear Autoregressive Neural Network Model

The NARNN model [40] is a kind of neural network with a memory function. The NARNN consists of an input layer, hidden layers and an output layer. The outputs of the network depend on the current input and the past output. The formula is expressed by:

(6) y(t)=f[y(t−1);⋯y(t−d)],

where y(t) denotes the outputs, *d* is the delay lag and *f* represents the nonlinear function.

To avoid over-fitting, the original data are often divided into a training set and a testing set. The number of delaying lags and hidden layer neurons is determined by repeated fitting. Finally, the model with good performance is selected.

#### 3.3.3. Exponential Smoothing Method

ES is a simple and effective forecasting method, which was proposed by Charles in 1957 [41]. The ES formula can be expressed as follows:

(7) $${\hat{x}}_{t+1}=\alpha x_{t}+\left(1-\alpha \right){\hat{x}}_{t},$$

where ${\hat{x}}_{t+1}$ is the forecast for the period t+1, $x_{t}$ is the observed value of series in period *t*, ${\hat{x}}_{t}$ is the forecast for the period *t* and α is the smoothing parameter between zero and one. If the time series is stable, then we select a small value of α. A large value of α is desired for non-stationary time series.

### 3.4. Combined Forecasting Model

To improve accuracy and diversify the risk of forecasting effectively, combined foresting is utilized to predict the IMFs and residue series.

Generally, the combining method can be expressed in the following form:

(8) $${\hat{x}}_{t}=\sum_{i=1}^{m}w_{i}x_{it},\;t=1,\;2,\;\cdots N,$$

where the weight $w_{i},\;i=1,2,\;\cdots ,m$ meets the conditions that $w_{i}\in \left[0,\;1\right],\sum_{i=1}^{m}w_{i}=1$.

In order to realize the combination forecasting, how to determine the weight $w_{i}$ is a key issue. The simple arithmetic method assigns an equal weight $w_{i}=1/m$ to each weight. In practice, the weighted average (WA) method has been shown to be an efficient tool for improving the accuracy of combination forecasting, which assigns different values to diverse weights according to the optimization method by minimizing the combination forecasting errors under the constraints that $w_{i}\in \left[0,\;1\right],\sum_{i=1}^{m}w_{i}=1$. It can be shown as follows:

(9) $$\begin{array}{c} \min J=\sum_{t=1}^{N}e_{t}^{2}=\sum_{i=1}^{m}\sum_{j=1}^{m}w_{i}w_{j}\sum_{t=1}^{N}e_{it}e_{jt}\\ s.t.\left\{\begin{array}{c} \sum_{i=1}^{m}w_{i}=1\\ w_{i}\geq 0,\;\;\;i=1,2,\cdots ,m \end{array}\right. \end{array}$$

where $e_{it}$ represents the error of the *i*-th individual forecasting method, and it can be written as $e_{it}=x_{t}-x_{it}$; $e_{t}$ is denoted as the error of combined forecasting, which can be written as:

$$e_{t}=x_{t}-{\hat{x}}_{t}=x_{t}-\sum_{i=1}^{m}w_{i}x_{it}=\sum_{i=1}^{m}w_{i}\left(x_{t}-x_{it}\right)=\sum_{i=1}^{m}w_{i}e_{it}.$$

By solving the above optimization model, we can obtain the optimal weight vector of each single forecasting method.

In the traditional combined forecasting model, the weights are generally fixed in each individual forecasting model. In fact, the forecasting accuracy varies with time points and methods; therefore, the IOWA operator is introduced to construct the optimal combined forecasting model by minimizing the sum of error squares.

The main ideas of the combined forecasting model based on the IOWA operator are as follows: firstly, the forecasting results of different methods at the same point are rearranged by forecasting accuracy; then, aggregate the rearrangement series by the WA method.

Let $a_{it}$ denote the forecasting accuracy of the *i*-th individual forecasting method at *t* moment; it can be defined as:

(10) ait=1−(xt−xit)(xt−xit)xtxt,(xt−xit)(xt−xit)xtxt<10,(xt−xit)(xt−xit)xtxt≥1.

Then, the combination forecasting result can be described as:

(11) $${\hat{x}}_{t}^{\prime }=\sum_{i=1}^{m}{w^{\prime }}_{i}x_{a-index(it)},$$

where $x_{a-index(it)}$ represents the rearrangement series based on forecasting accuracy.

The optimal CFMbased on the IOWA operator can be shown as follows:

(12) $$\begin{array}{c} \min J^{\prime }=\sum_{t=1}^{N}{\left({e^{\prime }}_{t}\right)}^{2}=\sum_{i=1}^{m}\sum_{j=1}^{m}\sum_{t=1}^{N}{w^{\prime }}_{i}{w^{\prime }}_{j}{e^{\prime }}_{it}{e^{\prime }}_{jt}\\ \;\;s.t.\;\;\;\left\{\begin{array}{c} \sum_{i=1}^{m}{w^{\prime }}_{i}=1\\ {w^{\prime }}_{i}\geq 0,\;\;\;i=1,2,\cdots ,m \end{array}\right. \end{array}$$

The optimal CFM is used to forecast the IMFs and residue series.

## 4. Empirical Study and Discussion

To verify the effectiveness of the proposed hybrid forecasting model, the AQI series of Hefei is utilized as the sample data. The other forecasting methods, including the GRNN model, NARNN model, ES model, these models based on the EEMD technique with mirror method (i.e., EEMD-MM-GRNN, EEMD-MM-NARNN, EEMD-MM-ES) and the simple weighted combined forecasting based on the EEMD technique with mirror method (EEMD-MM-SAM), are also introduced for comparison. Section 4.1 introduces the performance metrics. The model test and model improvement are presented in Section 4.2. Section 4.3 reports and discusses the corresponding results.

### 4.1. Statistical Measures for Forecasting Performance

To measure the forecasting accuracy and effectiveness of different models, many evaluation metrics have been researched and employed [27], such as the sum of squared error (SSE), mean absolute error (MAE), mean absolute percentage error (MAPE) and root mean squared error (RMSE). SSE is used to show the total forecasting error of the proposed model. MAE and RMSE are employed to evaluate the mean magnitude of error between the real value and forecasted value. MAPE is utilized to reflect the validity of the forecasting model. For these four metrics, the smaller the index values, the better the model performance.

The SSE, MAE, MAPE and RMSE are respectively defined as:

(13) $$SSE=\sum_{t=1}^{N}{\left(x_{t}-{\hat{x}}_{t}\right)}^{2},$$

(14) $$MAE=\frac{1}{N}\sum_{t=1}^{N}\left|x_{t}-{\hat{x}}_{t}\right|,$$

(15) $$MAPE=\frac{1}{N}\sum_{t=1}^{N}\left|\frac{x_{t}-{\hat{x}}_{t}}{x_{t}}\right|,$$

(16) $$RMSE=\sqrt{\frac{1}{N}\sum_{t=1}^{N}{\left(x_{t}-{\hat{x}}_{t}\right)}^{2}},$$

where $x_{t}$ and ${\hat{x}}_{t}\left(t=1,2,\ldots ,N\right)$ are the real value and forecasting value at time *t*, respectively. *N* is the data size of the testing set.

Besides, the mean mode accuracy (MMA) is introduced in this study, which can reflect the forecasting accuracy of classes. It can be described as:

(17) $$MMA=\frac{1}{N}\sum_{t=1}^{N}\sum_{c=1}^{C}b_{ct},$$

where:

(18) $$b_{ct}=\left\{\begin{array}{c} 1,\;\;\;{\hat{x}}_{t}\in c\\ 0,\;\;\;{\hat{x}}_{t}\notin c \end{array}\right.,$$

C={1,2,⋯,6},c∈C represents the class of time series value based on Table 1.

### 4.2. Testing Method and Improvements of the Proposed Model

The Diebold–Mariano (DM) test is employed to prove the superiority of the proposed hybrid forecasting approaches statistically [42]. The DM test investigates the null hypothesis that the expected forecast accuracy in the target model *A* is equal to that in the benchmark model *B*. The loss function is set to the mean squared error, and the DM statistic can be defined as follows:

(19) $$S_{DM}=\frac{\bar{g}}{\sqrt{({\overset{⌢}{V}}_{\bar{g}}}/N)},$$

where:

$$\bar{g}=\frac{1}{N}\sum_{t=1}^{N}g_{t},\;g_{t}={\left(x_{t}-x_{A,t}\right)}^{2}-{\left(x_{t}-x_{B,t}\right)}^{2},$$

and:

$${\overset{⌢}{V}}_{g}=\gamma_{0}+2\sum_{t=1}^{\infty }\gamma_{t}(\gamma_{t}=\mathrm{cov}(g_{t+1},g_{t}))$$

where $\gamma_{0}$ is the variance of $g_{t}$ and $x_{A,t},x_{B,t}$ represent the forecast values of model *A* and model *B* in period *t*, respectively. *N* is the number of observations in the test set. Here, a unilateral test is used to test the $S_{DM}$ statistic. Thus, the null hypothesis can be used to verify the superiority of model *A* over model *B* under the condition of accepting confidence level *p*.

In this study, an improvement rate is adopted to measure whether the model *A* is superior to model *B* in terms of forecasting accuracy. It can be defined as:

(20) $$IR_{RMSE}=\left|\frac{RMSE_{A}-RMSE_{B}}{RMSE_{A}}\right|,$$

where $RMSE_{A}$ and $RMSE_{B}$ represent RMSE values of the proposed model and competing model, respectively. According to (20), the bigger the value of $IR_{RMSE}$, the more superior the proposed model in forecasting.

The effectiveness of the forecasting model could be measured not only through the above statistical measures, but also could be described by the correlation coefficient; the correlation coefficient of the *i*-th method can be calculated by:

(21) Ri=∑t=1N(xt−x¯t)(x^t−^¯xt)∑t=1N(xt−x¯t)2∑t=1N(x^t−^¯xt)2,

where $x_{t}$ and ${\hat{x}}_{t}\;\;\left(t=1,2,\cdots ,N\right)$ are the real value and forecasting value at time *t*, respectively. *N* is the data size of the testing set, and ${\bar{x}}_{t}$ and ^¯xt are the mean of $x_{t}$ and ${\hat{x}}_{t}$, respectively.

### 4.3. Empirical Results

#### 4.3.1. Data Decomposition

The first step of the proposed hybrid forecasting model is to decompose the data of AQI in Hefei via the EEMD with MM. In the EEMD model, the ensemble member is set to 100, and the standard deviation of the added white noise is set to 0.05. Through the decomposition process, the time series of AQI in Hefei can be decomposed into a total of nine modes, i.e., eight IMFs and one residue.

**Figure 3:**
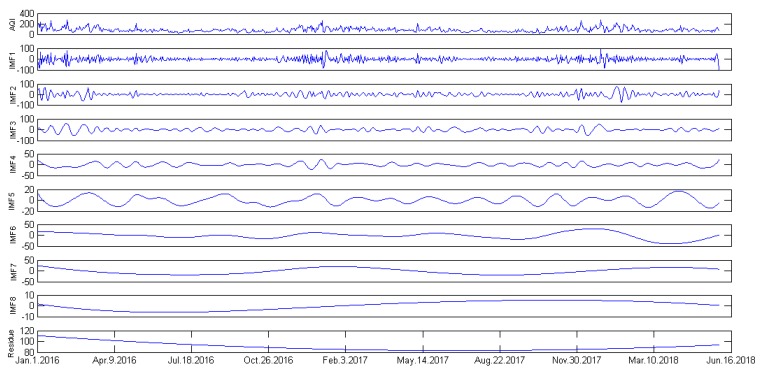
Data decomposition results of AQI in Hefei.

As shown in Figure 3, IMFs are listed in the order from the highest frequency to the lowest frequency, and the last one is the residue, which presents the trend of the AQI time series, and in reality, the AQI is closely related to seasonal factors such as temperature, radiation levels, humidity, precipitation, etc.

#### 4.3.2. Forecasting Results

**Figure 4:**
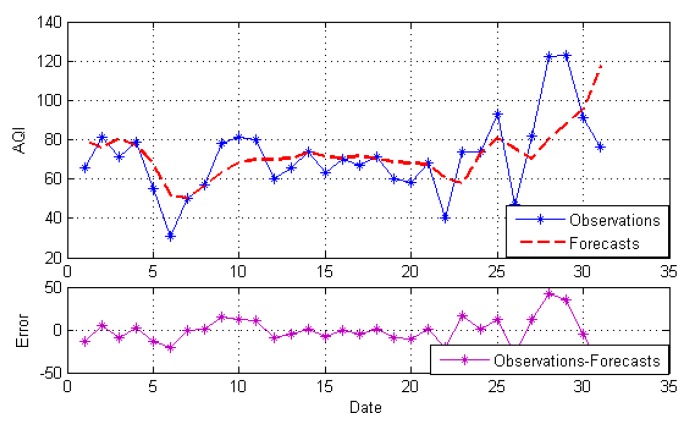
The forecasts and error based on EEMD-MM-GRNN.

**Figure 5:**
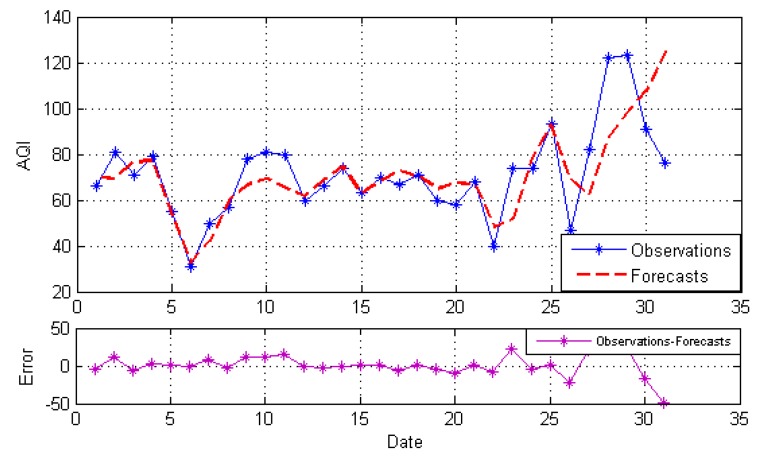
The forecasts and error based on EEMD-MM-NARNN.

**Figure 6:**
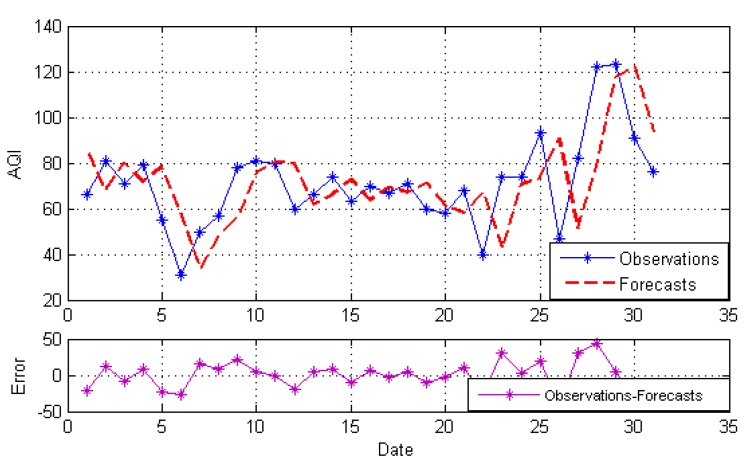
The forecasts and error based on EEMD-MM-ES.

**Figure 7:**
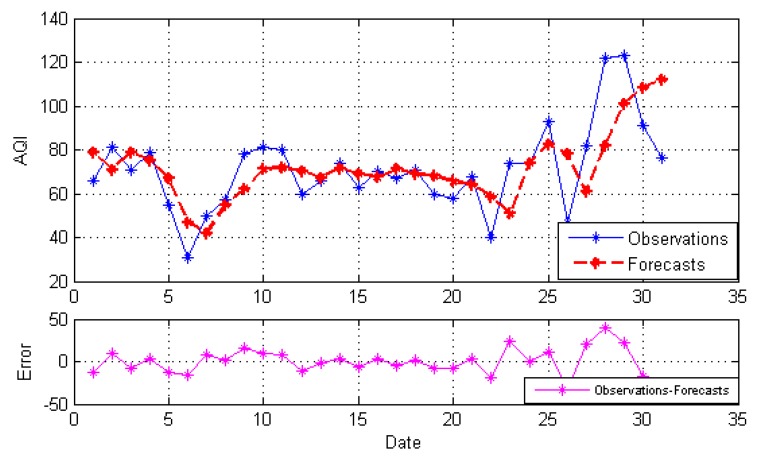
The forecasts and error based on EEMD-MM-SAM.

**Figure 8:**
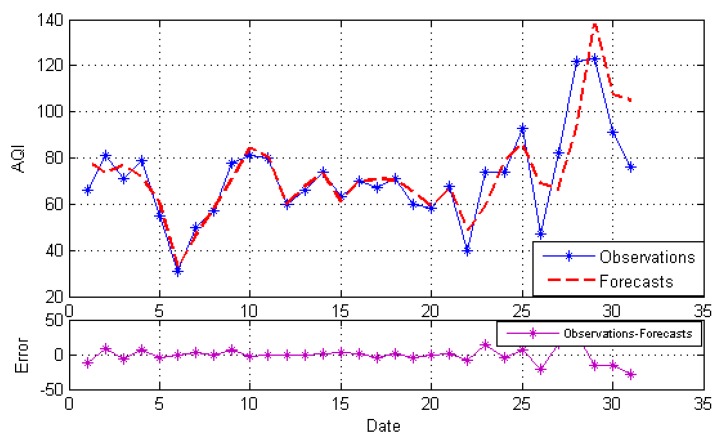
The forecasts and error based on EEMD-MM-CFM.

After decomposition, the GRNN model, NARNN model and ES model are employed to forecast the IMFs and the residue as the individual model, and then, the optimal combined forecasting model based on the IOWA operator is applied to obtain the combined results of IMFs and the residue. At last, the simple addition approach is used to combine the forecasting results of all modes into an ensemble result. The result is named EEMD-MM-CFM. For comparison, we just use the simple addition approach to combine individual forecasting and the simple arithmetic combined results of all modes. The results are named EEMD-MM-GRNN, EEMD-MM-NARNN, EEMD-MM-ES and EEMD-MM-SAM, respectively. Figure 4, Figure 5, Figure 6, Figure 7 and Figure 8 illustrate the different results according to the different models.

Figure 4, Figure 5 and Figure 6 report the forecasting results of three different individual models, respectively. As shown in Figure 4, Figure 5 and Figure 6, the forecasting performance of EEMD-MM-NARNN is the best; EEMD-MM-GRNN is inferior to the EEMD-MM-NARNN method; and EEMD-MM-ES has poor performance. However, the forecasting values of different methods at the same point are different according to the error. Therefore, we introduce the combined forecasting model by aggregating the effective information provided by different models.

Figure 7 and Figure 8 indicate the forecasting results of EEMD-MM-SAM and EEMD-MM-CFM. As shown in Figure 7, the EEMD-MM-SAM model performance is better than most of the individual models, but it is not exactly feasible in the domain of all the points. Hence, we introduce the IOWA operator and construct the optimal model for AQI forecasting. Figure 8 shows that the EEMD-MM-CFM model is superior to individual models and the EEMD-MM-SAM model for AQI forecasting in Hefei.

#### 4.3.3. Forecasting Performance Comparisons

**Figure 9:**
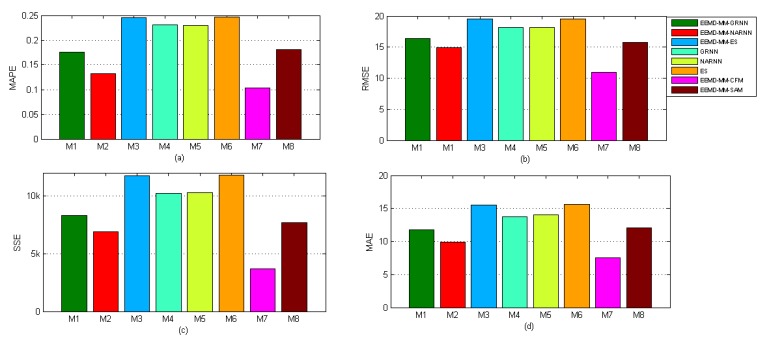
Performance comparison of different models in terms of different evaluation metrics.

**Figure 10:**
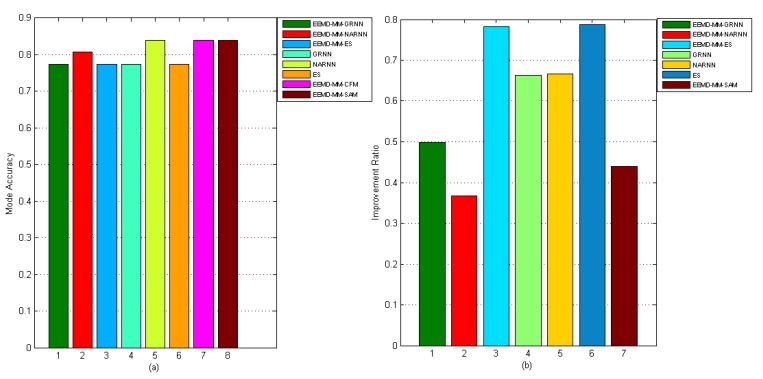
Performance comparison of different models in terms of MMA and IR.

The proposed hybrid forecasting model, i.e., EEMD-MM-CFM, and their seven benchmark models, including EEMD-MM-GRNN, EEMD-MM-NARNN, EEMD-MM-ES, GRNN, NARNN, ES and EEMD-MM-SAM, were used to forecast the AQI in Hefei. The performance comparison results are given in Figure 9 and Figure 10. From the results, we can obtain that the proposed model can be statistically proven to outperform all considered benchmark models in AQI forecasting in Hefei. In particular, the proposed model does not only get the lowest error, but also achieves high mode accuracy. Furthermore, the DM test statistically verifies the superiority of the proposed model over all benchmark models under the confidence level of 95%.

As for forecasting accuracy, the parts a, b, c and d in Figure 9 are represent the MAPE, RMSE, SSE and MAE criteria across different models for AQI data in Hefei, respectively. From Figure 9, we can obtain four important conclusions. First, the proposed model (EEMD-MM-CFM) performs significantly better than all considered benchmark models in AQI forecasting in Hefei, and the values of MAPE, RMSE, SSE and MAE are 0.1035, 10.93, 3702.8 and 7.52, respectively. Hence, the proposed technique can be applied as an effective method for AQI data analysis and forecasting in Hefei. Second, when comparing the proposed model with individual models (i.e., GRNN, NARNN and ES), the values of MAPE, RMSE, SSE and MAE are far smaller the individual models. Third, when comparing the proposed model with decomposition ensemble models (i.e., EEMD-MM-GRNN, EEMD-MM-NARNN and EEMD-MM-ES), the proposed model achieves the highest forecasting accuracy. The results prove that the proposed combined model is effective. Fourth, when comparing the proposed model with the simple arithmetic combined model (i.e., EEMD-MM-SAM), the weights of the individual forecasting models of which are equal, the proposed model is superior to the EEMD-MM-SAM model. The main reason is that the proposed model considers the forecasting accuracy of different models varying with time. Accordingly, the proposed model is an effective tool for AQI forecasting.

Regarding mode accuracy, the MMA results are presented in the part a in Figure 10, and similar conclusions can be found. The MMA of the proposed model is better than the EEMD-MM-GRNN, EEMD-MM-NARNN, EEMD-MM-ES, GRNN and ES models and is equal to the NARNN and EEMD-MM-SAM models. From the part b in Figure 10, we can see that the proposed model is an improvement over the other benchmark models. For instance, the maximum improvement percentage is for the ES model (78.72%), and the minimum improvement percentage is for the EEMD-MM-NARNN model (36.64%). Meanwhile, the correlation coefficient results are presented in Table 2. From Table 2, we can obtain three important conclusions. First, the proposed model (EEMD-MM-CFM) performs significantly better than all considered benchmark models. Second, when comparing the individual models (i.e., ES, NARNN and GRNN) with the according decomposition-ensemble models (i.e., EEMD-MM-ES, EEMD-MM-NARNN and EEMD-MM-GRNN), the values of the correlation coefficient corresponding to decomposition-ensemble models are larger than the individual models. Third, when comparing the proposed model with the simple arithmetic combined model (i.e., EEMD-MM-SAM), the proposed model is superior to the EEMD-MM-SAM model. Accordingly, the proposed model is an effective tool for AQI forecasting.

Furthermore, DM tests were employed for statistical demonstration, as the $S_{DM}$ statistics were listed with a *p*-value, shown in Table 3. Through the DM test, generally speaking, the proposed hybrid forecasting model outperforms the EEMD-MM-GRNN and EEMD-MM-NARNN models at a 5% level of statistical significance; and outperforms the EEMD-MM-ES, GRNN, NARNN and ES models at a 1% level of statistical significance. The results indicate that the proposed model can be statistically verified as significantly better than the other benchmark models, and it is proven again that the proposed model is an effective model for AQI forecasting.

## 5. Conclusions

This study proposed a hybrid forecasting approach by integrating EEMD based on the mirror method and the variable weighted combined forecasting model based on the IOWA operator for sub-series forecasting. The main steps of the proposed model are as follows: applying EEMD with the mirror method to sift the original AQI time series; then, the combined forecasting model was applied to forecast the IMFs and residue; finally, the outputs were obtained by summing the forecasts. In order to verify the effectiveness of the proposed model, four statistical measures including SSE, MAE MAPE and RMSE, as well as mode accuracy and the DM test were utilized. An example of the AQI forecasting in Hefei was illustrated to show that the effectiveness of the proposed method is guaranteed.

There are many individual forecasting models for AQI data. I this study, we only selected three individual models and combined them for the final output. We could introduce the optimal sub-models’ selection algorithm for a combined individual model in the future [43]. Furthermore, besides the AQI data, the proposed model should be extended to other forecasting tasks to test its generalization.

## Figures and Tables

**Table 1:** The AQI index and related information in China.

AQI	AQI Classes	Health Impact	Suggestions
0∼50	Excellent	The air quality is satisfactory	It is suitable for normal actions for various people.
51∼100	Good	Have weak health effects on extremely sensitive people	Extremely sensitive people should reduce outdoor activities.
101∼150	Light pollution	Healthy people show signs of irritation	Children, the elderly and patients with heart disease should reduce outdoor activities.
151∼200	Moderate pollution	It may affect the heart and respiratory systems of healthy people	Even healthy people should reduce outdoor sports activities.
201∼300	Serious pollution	The symptoms of heart disease and lung disease increased significantly	Children, the elderly and patients with heart disease should stop outdoor activities.
201∼300	Heavy pollution	Healthy people have obvious strong symptoms	Healthy people should avoid outdoor activities.

**Table 2:** The correlation coefficient of the proposed model.

Model	ES	NARNN	GRNN	EEMD-MM-SAM
Correlation Coefficient	0.4553	0.4663	0.4163	0.5988
Model	EEMD-MM-ES	EEMD-MM-NARNN	EEMD-MM-GRNN	EEMD-MM-CFM
Correlation Coefficient	0.4563	0.6754	0.5335	**0.8404**

**Table 3:** DM test results across different models.

Target Model	Benchmark					
	EEMD-MM-GRNN	EEMD-MM-NARNN	EEMD-MM-ES	GRNN	NARNN	ES
EEMD-MM-CFM	−2.059	−1.972	−3.027	−4.057	−2.972	−3.027
	(0.039)	(0.046)	(0.002)	(0.000)	(0.003)	(0.002)
EEMD-MM-GRNN		2.788	−1.455	−0.886	−0.681	−1.479
		(0.005)	(0.148)	(0.376)	(0.496)	(0.139)
EEMD-MM-NARNN			−1.617	−1.325	−0.979	−1.641
			(0.106)	(0.185)	(0.328)	(0.100)
EEMD-MM-ES				1.042	1.238	−1.884
				(0.297)	(0.216)	(0.050)
GRNN					−0.052	−1.064
					(0.958)	(0.287)
NARNN						−1.246
						(0.213)

## Abbreviations

AQI	Air quality
EEMD	Ensemble empirical mode decomposition
IMF	Intrinsic mode function
ANN	Artificial neural networks
MLR	Multiple linear regression
PCR	Principal component regression
SVR	Support vector regression
GRNN	General regression neural network
IOWA	Induced ordered weighted averaging
NARNN	Nonlinear autoregressive neural network
ES	Exponential smoothing
MM	Mirror method
CFM	Combined forecasting model
MMA	Mean mode accuracy
DM test	Diebold–Mariano test
IR	Improvements of the proposed model
SSE	Sum of squared error
MAE	Mean absolute error
MAPE	Mean absolute percentage error
RMSE	Root mean squared error
